# P-Type Pentatricopeptide Repeat Proteins YS1 and YS2 Function in Splicing of *petB* Intron to Maintain Chloroplast Homeostasis During Rice Seedling Development

**DOI:** 10.3390/ijms26094459

**Published:** 2025-05-07

**Authors:** Hui Sun, Yanshen Nie, Li Yu, Xiaohong Yue, Xin Hou, Jie Zhao

**Affiliations:** State Key Laboratory of Hybrid Rice, College of Life Sciences, Wuhan University, Wuhan 430072, China; sunhui123@whu.edu.cn (H.S.); nie_ys@whu.edu.cn (Y.N.); 2012102040065@whu.edu.cn (L.Y.); xiaohongyue@whu.edu.cn (X.Y.); xinhou@whu.edu.cn (X.H.)

**Keywords:** rice, pentatricopeptide repeat, chloroplast development, RNA splicing, photosystem protein

## Abstract

Regulating chloroplast gene expression is crucial for maintaining chloroplast function and plant development. Pentatricopeptide repeat (PPR) proteins form a vast protein family that regulates organelle genes and has multiple functions during plant development. Here, we found that two P-type PPR proteins, YS1 (yellow-green seedling 1) and YS2, jointly regulated seedling development in rice. The loss of *YS1* and *YS2* exhibited the collapsed chloroplast thylakoids and decreased photosynthetic activity, leading to the yellowing and death of rice seedlings. YS1 and YS2 could directly bind to the transcript of the *psbH-petB* intergenic region to facilitate the splicing of *petB* intron, thereby affecting the splicing efficiency of *petD*, which is located downstream of *petB* in the five-cistronic transcription unit *psbB-psbT-psbH-petB-petD*. The mutations in *YS1* and *YS2* led to decreased mature transcripts of *petB* and *petD* after splicing, significantly reducing the protein levels of PetB and PetD. This further led to deficiencies in the cytochrome b6/f and photosystem I complexes of the electron transport chain (ETC), ultimately resulting in decreased ETC-produced NADPH and reduced contents of carbohydrates in *ys* mutants. Moreover, transcriptome sequencing analysis revealed that YS1 and YS2 were vital for chloroplast organization and carbohydrate metabolism, as well as chloroplast RNA processing. In previous studies, the mechanism of *petB* intron splicing in the five-cistronic transcription unit *psbB-psbT-psbH-petB-petD* of rice is unclear. Our study revealed that the two highly conserved proteins YS1 and YS2 were functionally redundant and played critical roles in photosynthesis and seedling development through their involvement in *petB* intron splicing to maintain chloroplast homeostasis in rice. This work broadened the perspective on PPR-mediated chloroplast development and laid a foundation for exploring the biofunctions of duplicated genes in higher plants.

## 1. Introduction

Chloroplasts, which are organelles in photosynthetic organisms that capture light energy, have long been a subject of scientific curiosity for biologists in terms of their evolution and development [1]. Many structural, biochemical, and genomic data have supported the hypothesis of endosymbiotic origin, which suggests that prokaryotic cyanobacteria were engulfed by eukaryotes, not digested, but instead integrated into the host cells to become the current chloroplasts [2]. As the site of photosynthesis, chloroplasts are crucial for plant growth and development, and their dysfunction leads to serious defects in plants [3]. As semi-autonomous organelles, chloroplasts contain a complete genetic system that encodes more than 120 proteins. In general, chloroplasts contain more than 1000 proteins, most of which are encoded by nuclear genes. Therefore, the development of chloroplasts is regulated by their own genes and nuclear genes [4]. The regulation of chloroplast gene expression is extremely complex and includes RNA splicing, RNA editing, RNA translation, RNA cleavage, and RNA stabilization. Most of these processes require the involvement of nuclear-encoded proteins [5].

RNA splicing refers to the process of removing introns from pre-mRNA and joining exons together. Organelles contain group I and group II introns, the vast majority of which belong to group II [6]. There are 17 group II introns and 1 group I intron in rice and maize chloroplasts, and 20 group II introns and 1 group I intron in *Arabidopsis* chloroplasts [7]. Group II introns in plants have lost the ability to self-catalyze and need nuclear-encoded proteins as cofactors. The cofactors are mainly distributed in PPR (pentatricopeptide repeat) proteins, CRM (chloroplast splicing and ribosome maturation) proteins, RH (RNA helicase) proteins, and the PORR (plant organelle RNA recognition) and APO (apolipoprotein) families [8]. PPR proteins are a vital class of cofactors involved in regulating the splicing of many organelle transcripts.

PPR proteins, one of the largest protein families in land plants, are a class of RNA-binding proteins involved in the post-transcriptional processing of organelle genes. They have profound impacts on the biosynthesis and function of organelles [9]. PPR proteins are mainly divided into P types and PLS types. P-type PPR proteins are composed of 35 relatively conserved amino acids in a tandem and repeated arrangement, while PLS-type PPR proteins are arranged by the P motif (35 amino acids), L (longer) motif (35~36 amino acids), and S (shorter) motif (31~32 amino acids) as a repeating unit [10]. In addition, PLS-type members can be classified into E, E+, and DYW subclasses based on their different C-terminal amino acid sequences. The E and E+ domains contain 34 amino acids, a structure similar to that of the TPR (tetratricopeptide repeat) protein, while the DYW domain contains the cytidine deaminase sequence and is named after three conserved amino acids: aspartate (D), tyrosine (Y), and tryptophan (W) [11].

The PPR4 protein in maize and the OTP51 protein in *Arabidopsis* affect the splicing of the first intron of *rps12* gene and the second intron of *ycf3* gene, respectively [12,13]. In addition, both AtPPR4 and EMB2654 affect the trans-splicing of *rps12* gene [14,15]. OsWSL is a PPR protein localized in chloroplasts. In the *oswsl* mutant, the splicing of the chloroplast gene *rpl2* is severely hindered, resulting in an abnormal *rpl2* transcript and a defective RPL2 protein [16]. The *WSL4* gene in rice encodes a P-type PPR protein containing 12 PPR motifs, which is located in the chloroplast nucleoid. The leaves of *wsl4* mutant show white stripes, decreased chlorophyll content, and defective splicing of the chloroplast genes *atpF*, *ndhA*, *rpl2*, and *rps12* [17]. *WSL5* also encodes a PPR protein that targets chloroplasts. The mutation of *WSL5* causes white stripes during early leaf development and defects in the editing of *rpl2* and *atpA* and in the splicing of *rpl2* and *rps12* [18]. Moreover, the SOT5/EMB2279 protein in *Arabidopsis* is essential for the splicing of plastid genes *rpl2* and *trnK* [19]. OsPPR6 is a PLS-like PPR protein with 17 PPR motifs and a C-terminal DYW domain. The *osppr6* mutant exhibits early chloroplast development defects that failed to form thylakoid membranes, leading to leaf whitening and seedling death [20]. The *OspTAC2* gene encodes a chloroplast protein containing 10 PPR domains. The thylakoid membrane in the *osptac* mutant is irregular, and the OspTAC2 protein plays a crucial role in the chloroplast development of rice [21].

*psbB-psbT-psbH-petB-petD* is a five-cistronic transcription unit in chloroplasts that encodes five proteins of two thylakoid membrane complexes. *psbB*, *psbT*, and *psbH* encode subunits of photosystem II, while *petB* and *petD* encode core subunits of the cytochrome b6/f complex [22,23]. In *Arabidopsis*, HCF107 (high chlorophyll fluorescence 107) is involved in the intercistronic processing of the 5′ terminal *psbH* untranslated region and in stabilizing the processed 5′ terminal *psbH* RNA, thus affecting the assembly of PSII [24]. In maize, CRP1 (chloroplast RNA processing 1) binds to the *petB-petD* intergenic region and affects PetD synthesis; therefore, its mutation leads to a defective cytochrome b6/f complex and photosystem I in the *crp1* mutant [25]. The *osppr4* mutant exhibits an albino seedling phenotype and partial defects in the splicing of *petB* intron, as identified using RT-PCR [26]. OsSLA4 (seedling-lethal albino 4) is essential for chloroplast structure and seedling growth in rice. The splicing of the *petB* intron is affected in *sla4*, as detected using RT-PCR [27]. However, how OsPPR4 and OsSLA4 are involved in the splicing of the *petB* intron and their binding sites on the *petB* transcript remain unknown. Additionally, the key factors involved in the splicing of the *petB* intron in rice also need to be further investigated.

In this study, we obtained a rice mutant, *ys* (*yellow-green seedling*), from the rice T-DNA insertion sequence database, which exhibited seedling yellowing and, eventually, death. Sequence analysis revealed that *YS1* and *YS2* encode P-type PPR proteins containing 11 and 12 PPR motifs, respectively, and they were predominantly expressed in the leaves. Our research found that YS1 and YS2 directly bound to the transcript of the *psbH-petB* intergenic region and participate in the splicing of the *petB* and *petD* introns. Therefore, losses of these two genes led to decreased levels of the PetB and PetD proteins and reduced accumulation of the cytochrome b6/f complex and photosystem I protein in *ys1 ys2-cr* mutants, which ultimately caused the collapse of the thylakoid structure, decreased photosynthetic activity, and reduced chlorophyll content. Moreover, the contents of carbohydrates, such as glucose, sucrose, fructose, and starch, were decreased significantly in the mutant *ys* seedlings. Our research revealed that the PPR proteins YS1 and YS2 were functionally redundant and played critical roles in photosynthesis and seedling development by affecting *petB* intron splicing and chloroplast carbohydrate metabolism in rice.

## 2. Results

### 2.1. Phenotypic Features of ys Mutants

**Figure 1:**
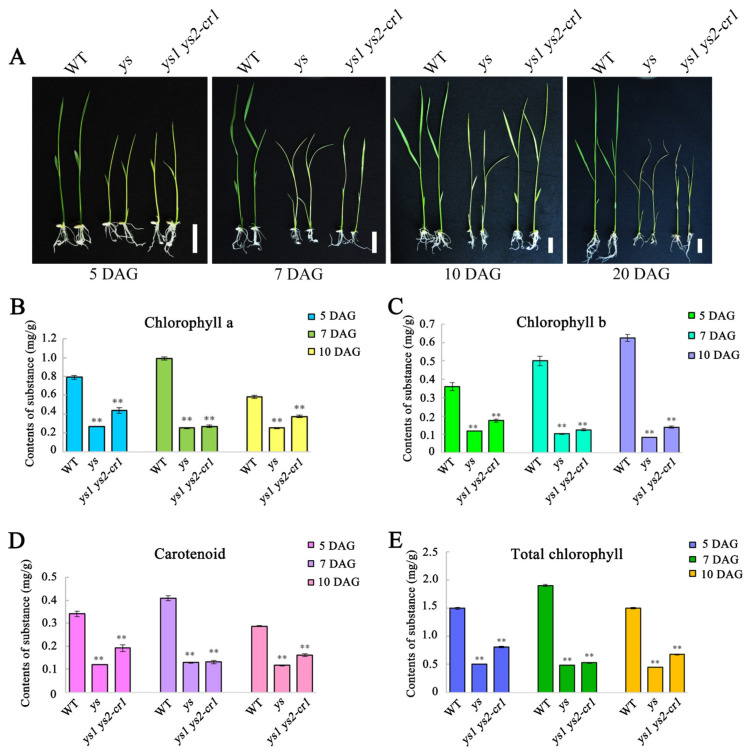
Phenotypic characteristics and chlorophyll contents of *ys* mutants in rice. (**A**) Seedlings at 5, 7, 10, and 20 days after germination (DAG) of WT, *ys* and *ys1 ys2-cr1*. Scale bars = 1 cm. (**B**) The contents of chlorophyll a in WT, *ys* and *ys1 ys2-cr1* seedlings at 5, 7 and 10 DAG. (**C**) The contents of chlorophyll b in WT, *ys* and *ys1 ys2-cr1* seedlings at 5, 7 and 10 DAG. (**D**) The contents of carotenoid in WT, *ys* and *ys1 ys2-cr1* seedlings at 5, 7 and 10 DAG. (**E**) The total chlorophyll contents in WT, *ys* and *ys1 ys2-cr1* seedlings at 5, 7 and 10 DAG. The values were calculated for three biological replicates and three technical replicates. ** means *p* < 0.01.

We obtained an *ys* mutant without the insertion of T-DNA from the RISE DB (Rice T-DNA Insertion Sequence Database). Previous studies in our lab have shown that the *ys* mutant might be controlled by double recessive genes YS1 and YS2, as determined through sequencing-based mapping [28]. By observing *ys* seedlings at 5, 7, 10, and 20 days after germination (DAG), we found that the *ys* seedlings etiolated and eventually died at about 20 DAG (Figure 1A). To verify that the phenotypic characteristics of the *ys* mutant were caused by *YS1* and *YS2* mutations, we designed the target sites (Figure S1; Figure S2A,B) and knocked out the two genes using the CRIPSR/Cas9 technique and the callus infection method. As a result, we obtained *ys1-cr*, *ys2-cr*, and *ys1 ys2-cr* mutant plants. Similar to *ys*, the double gene mutants *ys1 ys2-cr1* (Figure 1A) and *ys1 ys2-cr2* (Figure not shown) exhibited seedling yellowing and were eventually lethal, while the single gene mutants *ys1-cr1*, *ys1-cr2*, and *ys2-cr1* seedlings showed no difference from the wild type (WT) (Figure S2C–E). These results indicated that YS1 and YS2 were functionally redundant, and their mutations resulted in the yellowing and death of rice seedlings.

To research the changes in the chlorophyll contents of *ys* mutants, we identified the contents of chlorophyll a, chlorophyll b, carotenoid, and total chlorophyll in WT, *ys*, and *ys1 ys2-cr1* seedlings at 5, 7, and 10 DAG of rice. The results showed that the contents of chlorophyll a (Figure 1B), chlorophyll b (Figure 1C), carotenoid (Figure 1D), and total chlorophyll (Figure 1E) in *ys* and *ys1 ys2-cr1* were significantly reduced compared with the WT, suggesting that rice YS1 and YS2 might play critical roles in chloroplast development.

### 2.2. The Chloroplast Thylakoid Structure Was Collapsed in ys

**Figure 2:**
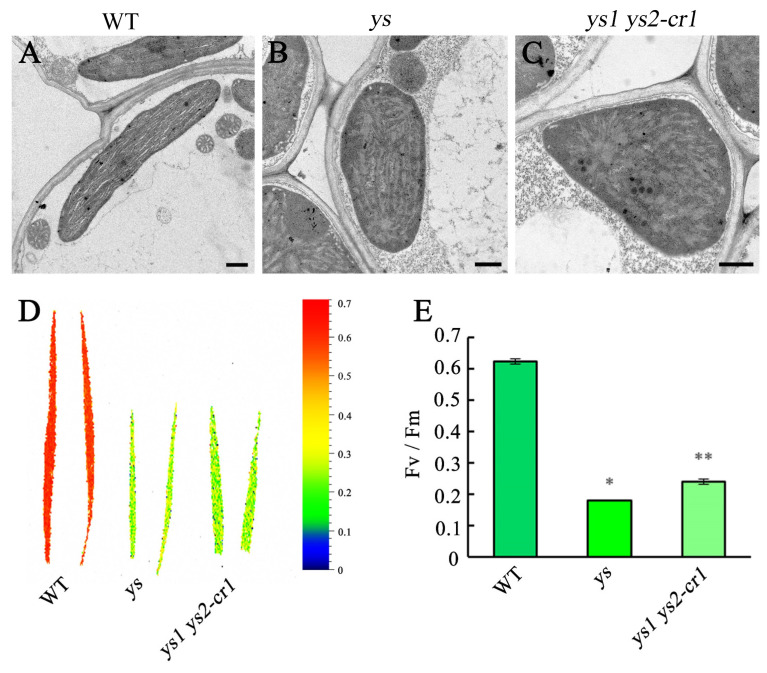
Chloroplast ultrastructure and photosynthetic efficiencies in WT, *ys* and *ys1 ys2-cr1* at 7 DAG leaves of rice. (**A**–**C**) Chloroplast ultrastructure. (**A**) In WT, the chloroplasts were long shuttle shaped, the grana of thylakoids were stacked in order, and the grana lamellae connected with each other. (**B**,**C**) The chloroplast shapes were irregular, and the thylakoid stacks were disorganized in *ys* and *ys1 ys2-cr1*. Scale bars = 0.5 mm in pictures A–C. (**D**,**E**) Photosynthetic efficiencies in 7 DAG leaves of WT, *ys* and *ys1 ys2-cr1*. (**D**) The maximum fluorescence (Fv/Fm) of 7 DAG leaves in WT, *ys* and *ys1 ys2-cr1*. The Chlorophyll fluorescence images were taken by instrument Fluor Cam800MF (Photon Systems Instruments Ltd., Brno, Czech Republic). The seedling leaves were cut and placed in square petri dishes containing 1/2 MS solid medium after 30 min dark adaption. The color scale ranging from dark blue (0) to red (0.7) represents Fv/Fm. (**E**) The values of Fv/Fm in 7 DAG WT, *ys* and *ys1 ys2-cr1* leaves. In (**E**), the values were calculated for four biological replicates. * means *p* < 0.05; ** means *p* < 0.01.

To explore whether chloroplasts in the mutants were affected, we observed the chloroplast ultrastructure of 7 DAG leaves from the WT, *ys*, and *ys1 ys2-cr1* using transmission electron microscopy. The chloroplasts in WT were long shuttle shaped, the grana of the thylakoids were arranged in an orderly stacked structure, and the grana lamellae were connected with each other (Figure 2A). Meanwhile, the chloroplast shapes in the *ys* and *ys1 ys2-cr1* were irregular, and the thylakoid stacks were disorganized (Figure 2B,C). By observing the mitochondria (Figure S3A–C), endoplasmic reticulum (Figure S3D–F), and cell membrane (Figure S3G–I), we found that the ultrastructures of these organelles in the *ys* and *ys1 ys2-cr1* were regular and ordered, with no difference from those in the WT. This indicated that rice YS1 and YS2 were essential for the development of chloroplast structure.

### 2.3. Photosynthetic Activity Was Reduced in ys

To investigate the photosynthetic efficiencies in *ys* mutants, we tested the maximum fluorescence (Fv/Fm) of 7 DAG leaves from the WT, *ys,* and *ys1 ys2-cr1*, which reflected their maximum photochemical efficiency. Our results showed that their maximum fluorescence values in the *ys* and *ys1 ys2-cr1* were decreased by approximately 3.5 and 2.6 times, respectively, compared to the WT (Figure 2D,E), indicating that the photosynthetic activities were evidently reduced in the mutants.

### 2.4. PSI and Cytochrome b6/f Complex Were Impaired in ys

**Figure 3:**
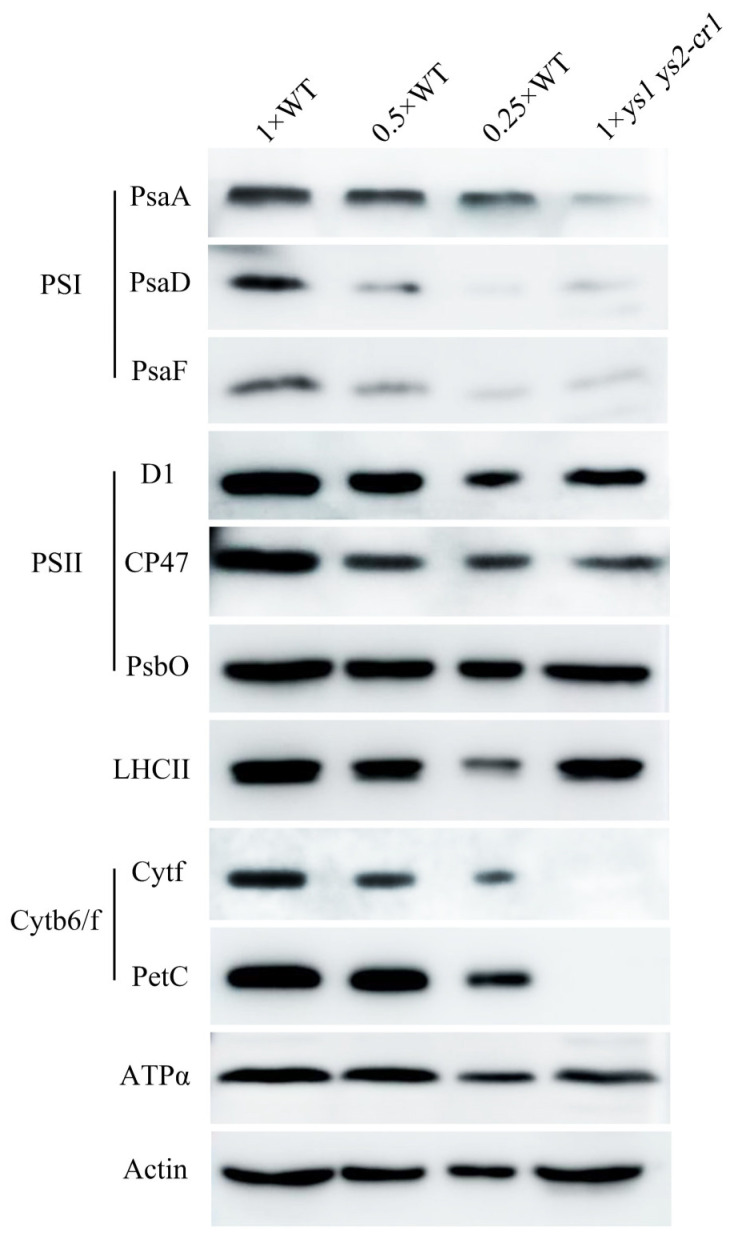
Immunoblot analysis of photosystem proteins in WT and *ys1 ys2-cr1* of rice. The PsaA, PsaD and PsaF are PSI subunits; D1, CP47 and PsbO are PSII subunits; Cytf and PetC are cytochrome b6/f complex subunits; LHCII is the light harvesting antenna protein of PSII; ATPα is the core subunit of ATP synthase. Actin was used as an internal reference for the consistency of sample loading. Primary antibodies against photosystem proteins and Actin were the Goat anti-Rabbit antiserums and the Goat anti-Mouse antiserum, respectively.

The photosynthetic system consists of photosystem I (PSI), photosystem II (PSII), the cytochrome b6/f complex, and ATP synthase [29]. By examining the levels of photosynthetic system proteins in the WT and *ys1 ys2-cr1*, we found that the protein levels of PSI subunits PsaA, PsaD, and PsaF, as well as the cytochrome b6/f complex subunits Cytf and PetC, were seriously decreased in *ys1 ys2-cr1*. Meanwhile, the protein levels of PSII subunits D1, CP47, and PsbO, and the ATP synthase core subunit ATPα were not different from those of the WT (Figure 3), suggesting that the accumulation of PSI and cytochrome b6/f complex proteins in *ys1 ys2-cr1* was inhibited, which might be one of the reasons for the decreased photosynthetic activity.

In addition, we detected protein expression levels of mitochondrial complex components. The results showed that the protein levels of respiratory chain complex I subunit NAD9, complex II subunit SdhA, complex III subunit UQCRC2, complex IV subunit COXII, complex V subunit ATP1, and electron acceptor Cyt c in *ys*, *ys1 ys2-cr1,* and *ys1 ys2-cr2* were no different from those in the WT (Figure S4). This indicated that the mitochondrial complex was not deficient in the *ys* mutants, implying that YS1 and YS2 did not function in mitochondria.

### 2.5. YS1 and YS2 Encode Two Highly Conserved P-Type PPR Proteins

*YS1* and *YS2* consist of 1632 and 2094 base pairs and encode 543 and 697 amino acids, respectively. Each has only one exon and no intron. The sequence of 543 amino acids in YS1 is almost entirely contained within the sequence of 697 amino acids in YS2, with the exception of two amino acids at sites 61 and 184 in YS1 (Figure S1). This indicated that YS1 and YS2 were two proteins with highly similar sequences. Based on predictions using the PPR website (https://ppr.plantenergy.uwa.edu.au), YS1 and YS2 contain 11 and 12 putative P-type PPR motifs, respectively (Figure S1), suggesting that YS1 and YS2 were both P-type PPR proteins.

We downloaded homologous sequences of YS1 and YS2 in 43 different species from NCBI, aligned the resulting 44 sequences using ClustalX software, and constructed a phylogenetic tree using MEGA7.0 software. Phylogenetic analysis showed that YS1 and YS2 were not plant-specific proteins and that their homologous sequences were widely distributed in dicotyledons, monocotyledons, liverwort, *Physcomitrella patens*, animals, and bacteria. They were most conserved in monocotyledons, such as *Brachypodium distachyon*, ryegrass, barley and wheat, and followed by dicotyledons, liverwort, *Physcomitrella patens*, animals and bacteria (Figure S5). We further aligned 20 homologous protein sequences of YS1 and YS2 from different monocotyledon species using DNAMAN software and found that these sequences were 76.28% similar (Figure S6A). The 20 proteins were predicted using the PPR website, and all were P-type PPR proteins with relatively conserved PPR motifs (Figure S6B). These results indicated that YS1 and YS2 in monocotyledons were highly conserved and might have similar functions.

### 2.6. YS1 and YS2 Were Predominantly Expressed in Leaves

To analyze the expression patterns of *YS1* and *YS2*, we downloaded their FPKM expression values from RNA sequencing data available in the RGAP database (http://rice.uga.edu). Our analysis found that both YS1 and YS2 were the most highly expressed in 20-day leaf, followed by the pistil, post-emergence inflorescence, pre-emergence inflorescence, 25 DAP embryo, and shoot, with low expression levels in other tissues (Figure S7A,B). In addition, we obtained the microarray expressions of *YS1* and *YS2* in specific tissues and organs at different developmental stages under growth conditions from the RiceXPro website (Available online: https://ricexpro.dna.affrc.go.jp/data-set.html (accessed on 24 October 2024)). The results displayed that *YS1* and *YS2* were predominately expressed in vegetative leaf, reproductive leaf and ripening leaf, while lightly expressed in the reproductive stem and ripening stem, 0.6–1.0 mm and 5.0–10 mm inflorescences, 5–10 cm and 10–14 cm pistils, and 14 DAF and 28 DAF embryos (Figure S7C). Moreover, we examined the mRNA expression levels of *YS1* and *YS2* in different tissues of the WT using an RT-qPCR assay. Since *YS1* is almost entirely included in *YS2*, only the expression of *YS2* and the co-expression of *YS1* and *YS2* could be specifically detected. We tested rice seedlings, roots, stems, leaves, and panicles at the P1 (panicle stage 1), P4, and P6 stages, pistil, stamens, and seeds. We found that *YS1* and *YS2* were expressed in all tested tissues, with the highest expression levels in the 60-day leaves (Figure S7D,E), suggesting that YS1 and YS2 might play a crucial role in rice leaves.

### 2.7. petB and petD Intron Splicing Were Impeded in ys Mutants

**Figure 4:**
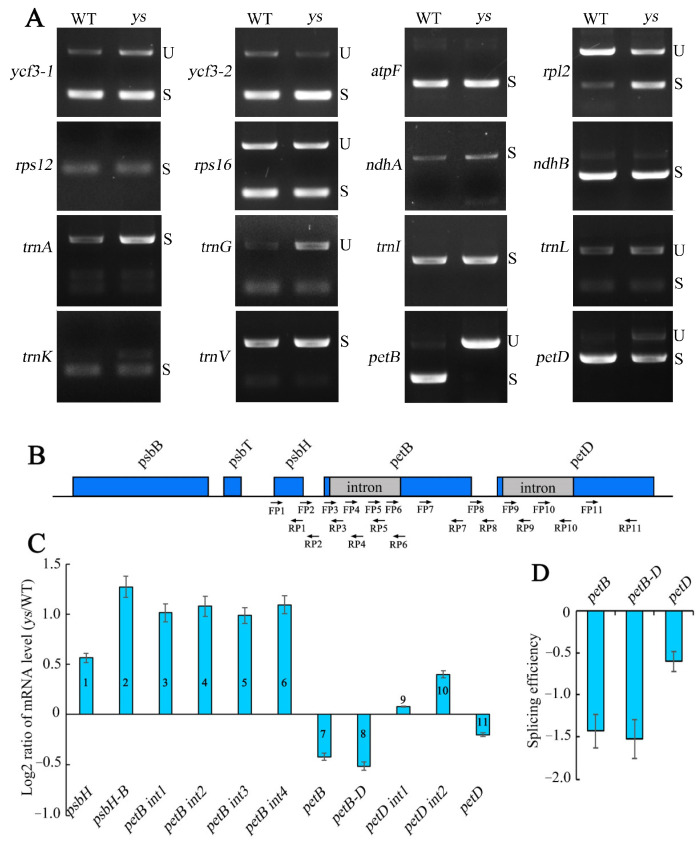
Chloroplast RNA splicing analysis in WT and *ys*. (**A**) Splicing of chloroplast introns detected by semi-quantitative PCR assay. U: unsplicing; S: splicing. (**B**) Structure diagrams of five-cistronic transcription unit *psbB-psbT-psbH-petB-petD* in rice. The blue boxes and gray boxes represent exons and introns, respectively, and the black lines between genes represent intergenic regions. The black arrows represent the location of the primers used for RT-qPCR assay in picture C. (**C**) RT-qPCR analysis of *petB*, *petD* and their adjacent transcripts in WT and *ys* leaves at 7 DAG. The numbers on the columns correspond to the used primer numbers marked in picture B. (**D**) The splicing efficiency of *petB* and *petD*. Splicing efficiency = Log_2_[(Spliced *ys*/unspliced *ys*)/(Spliced WT/unspliced WT)]; the primers used on *petB*, *petB-D* and *petD* are primer pairs 7 and 3, 8 and 3, 11 and 9 marked in picture B, respectively. The values were calculated for three biological replicates and three technical replicates.

To explore the effects of YS1 and YS2 on chloroplast intron splicing, we examined the splicing of chloroplast introns in the WT and *ys* mutants with a semi-quantitative PCR assay using primers on both sides of the introns. The results showed that there was almost no spliced short fragment in the amplification products of the *petB* introns in the *ys* compared to the WT, suggesting that the introns of *petB* could not be spliced (Figure 4A). Additionally, the semi-quantitative PCR products of *petD* contained both unspliced full-length intron fragments and spliced short fragments in the *ys*, indicating that the splicing efficiency of the *petD* intron was decreased (Figure 4A). *petB* and *petD* are two parts of the five-cistronic transcription unit *psbB-psbT-psbH-petB-petD* (Figure 4B). To verify the above results of intron splicing, we designed primers that cross introns and on introns to examine the splicing efficiency of transcripts. The splicing efficiency was calculated as Log_2_[(Spliced *ys*/unspliced *ys*)/(Spliced WT/unspliced WT)] using an RT-qPCR assay. Our results displayed that the mRNA levels and splicing efficiencies of *petB* and *petD* were decreased in the *ys* compared to the WT (Figure 4C,D), suggesting that YS1 and YS2 were key factors in the splicing of the *petB* and *petD* transcripts.

### 2.8. YS1 and YS2 Directly Bound to the psbH-petB Intergenic Region Transcript

**Figure 5:**
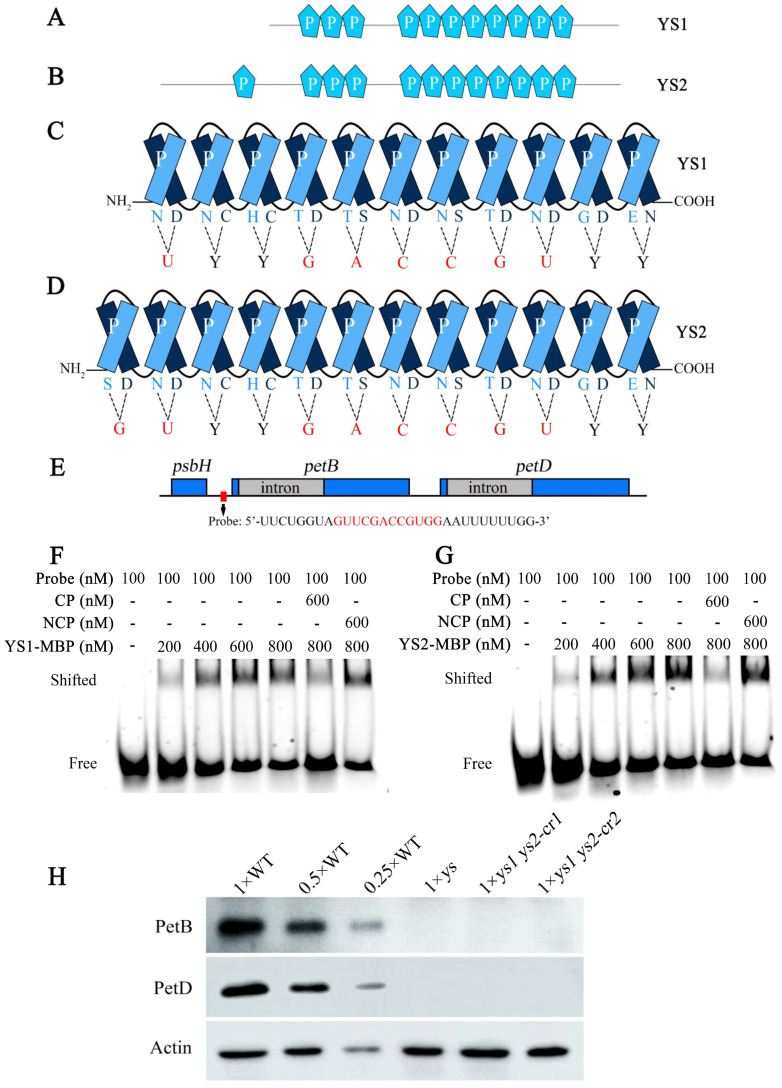
YS1 and YS2 bound to chloroplast target RNA. (**A**) Structural diagram of rice YS1. (**B**) Structural diagram of rice YS2. The light blue irregular pentagons represent PPR motifs. There were 11 and 12 PPR motifs in YS1 and YS2 sequences, respectively. (**C**) The predicted recognition codes of PPR motifs in YS1 protein. (**D**) The predicted recognition codes of PPR motifs in YS2 protein. “Y” represents C or U. The PPR motif is a helical-loop-helical structure, and the 5th amino acid and the 35th amino acid of a motif together recognized a binding RNA code. The bases marked in red are the codes recognized by PPR motifs in YS1 and YS2. (**E**) The schematic diagram of RNA probe position. Red box represents the probe position, and the red sequence is the predicted recognition codes. The 5′ end of RNA probe is labeled with FAM fluorophore. (**F**) RNA electrophoretic mobility shift assay (REMSA) of YS1 protein and *psbH-petB* intergenic RNA. (**G**) REMSA of YS2 protein and *psbH-petB* intergenic RNA. CP: competitive probe; NCP: noncompetitive probe; The CP sequence: UUCUGGUAGUUCGACCGUGGAAUUUUUUGG; The NCP sequence: UUCUGGUACAAGCUGGCACCAAUUUUUUGG; The bases in CP sequences marked in red are the predicted recognition codes, and the red bases in NCP sequence are the mutant codes. The two ends of the probe, CP and NCP sequences are native protective bases with black color. (**H**) Protein levels of PetB and PetD in WT, *ys*, *ys1 ys2-cr1* and *ys1 ys2-cr2* leaves at 7 DAG. The PetB and PetD are cytochrome b6/f complex subunits. Primary antibodies against PetB and PetD used in the immunoblot were the Goat anti-Rabbit antiserums.

YS1 and YS2 were two P-type PPR proteins containing 11 and 12 PPR motifs, respectively (Figure S1; Figure 5A,B). According to the prediction of PPR recognition code [30], we identified the potential target sites for YS1 and YS2 on the transcript of the *psbH-petB* intergenic region and designed an FAM-labeled RNA probe (Figure 5C–E). Because PPR proteins can directly bind to target RNA, we performed an RNA electrophoretic mobility shift assay (REMSA). The results showed that the band migration rates of the protein–RNA complexes were slower than the free RNA probe, and the band migration intensity was increased with increasing YS1 and YS2 proteins. When a sufficient competitive probe (CP) was provided, the migrated band weakened; when a sufficient noncompetitive probe (NCP) was provided, the migrated band remained unchanged (Figure 5F,G), which indicated that the YS1 and YS2 proteins could directly bind to the RNA of the *psbH-petB* intergenic region. Since *petB* and *petD* are two downstream parts of the five-cistronic transcription unit *psbB-psbT-psbH-petB-petD*, we examined their protein levels in WT and *ys* mutants. The results displayed that the PetB and PetD protein levels were barely detectable in the *ys*, *ys1 ys2-cr1,* and *ys1 ys2-cr2* mutants compared to WT (Figure 5H). These results suggested that YS1 and YS2 directly bound to the transcript of the *psbH-petB* intergenic region and participate in the splicing of the *petB* intron. This affects the splicing efficiency of *petD*, which is downstream of *petB* in the transcription unit *psbB-psbT-psbH-petB-petD*. Ultimately, the loss of YS1 and YS2 led to a decrease in the translation of PetB and PetD proteins in the *ys* mutants.

### 2.9. Photosynthetic Electron Transfer Was Damaged in ys Mutants

Ferredoxin-NADP^+^ oxidoreductase (FNR) is the terminal electron carrier in the photosynthetic electron transfer chain. FNR mediates electron transfer from ferredoxin (Fd) to NADP^+^ and produces NADPH according to the following reaction: 2Fd (reduced state) + NADP^+^ + H^+^→ 2Fd (oxidized state) + NADPH. NADPH is mainly used for CO_2_ fixation in the Calvin cycle [31,32]. Based on our results, the key electron carrier cytochrome b6 (PetB) protein in the photosynthetic electron transfer chain was deficient, leading to impaired cytochrome b6/f complex and PSI, which are involved in the electron transport process, in the *ys*. To determine whether the expression level of the terminal electron carrier FNR was changed in *ys*, we detected the FNR1 protein levels in WT and *ys* mutants using an immunoblot assay. The results showed that the FNR1 protein levels in the *ys*, *ys1 ys2-cr1*, and *ys1 ys2-cr2* were reduced and retained less (Figure S8A). Moreover, we measured the NADPH concentration in WT and *ys* mutants and found that the *ys*, *ys1 ys2-cr1*, and *ys1 ys2-cr2 mutants* displayed significant decreases compared to WT (Figure S8B). These results indicated that the photosynthetic electron transfer in the *ys* was disrupted, which might affect carbon fixation during photosynthesis, revealing the crucial roles of YS1 and YS2 in the chloroplast electron transfer chain.

### 2.10. Altered Expression Profile in ys Mutants

**Figure 6:**
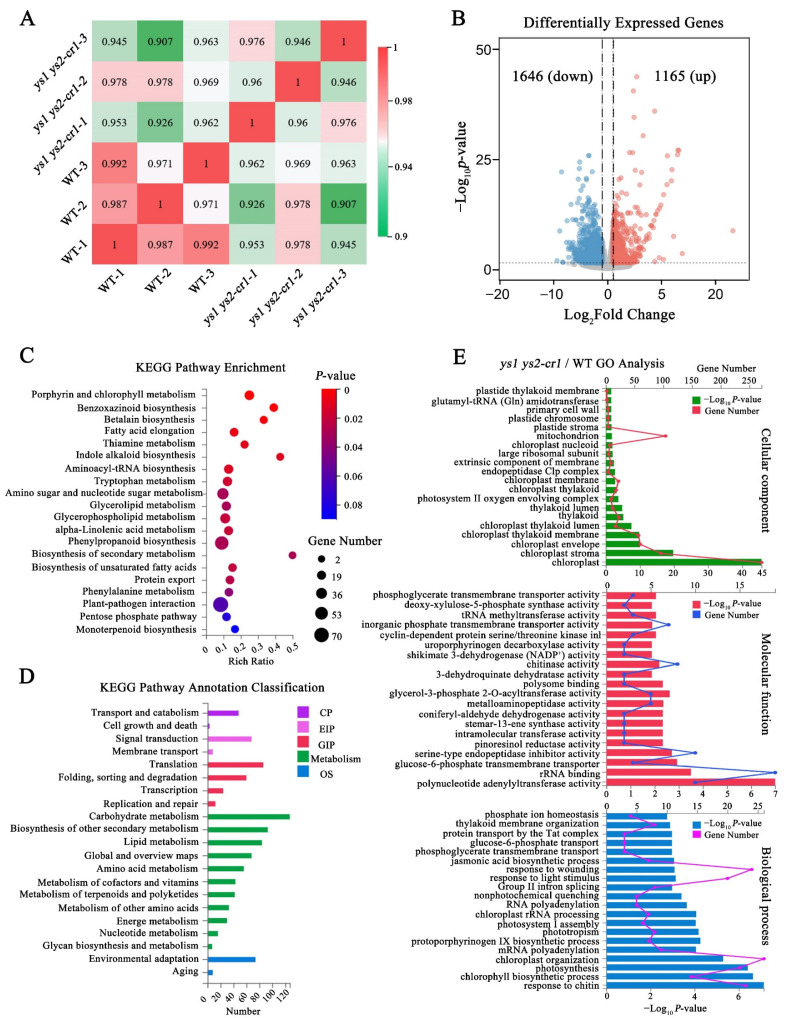
GO (Gene Ontology) and KEGG (Kyoto Encyclopedia of Genes and Genomes) enrichment analysis in the transcriptome of WT and *ys1 ys2-cr1*. (**A**) Correlation analysis of transcriptome data between WT and *ys1 ys2-cr1*. The higher the correlation coefficient between samples, the more similar the gene expression levels. (**B**) Differentially expressed genes between WT and *ys1 ys2-cr1*. The blue dots represent down-regulated genes, the red dots represent up-regulated genes, and the gray area in the middle represents not significantly changed genes. (**C**) The KEGG enrichment map of differential genes between WT and *ys1 ys2-cr1*. The differential genes were analyzed based on three biological replicates with |log_2_ (fold change)| ≥ 1. (**D**) The KEGG pathway annotation classification of differential genes between WT and *ys1 ys2-cr1*. CP: Cellular Processes; EIP: Environmental Information Processing; GIP: Genetic Information Processing; OS: Organismal Systems. (**E**) The GO enrichment map of differential genes between WT and *ys1 ys2-cr1*. The GO analysis consists of cellular component, molecular function and biological process.

To further investigate the impacts of YS1 and YS2 on rice, we performed transcriptome sequencing of WT and *ys1 ys2-cr1* seedlings at 7 DAG. Correlation analysis of the gene expressions between the samples (Figure 6A) and volcano plot analysis of differential gene expressions (Figure 6B) showed that the gene expression profile in *ys1 ys2-cr1* was significantly different from that of the WT. There were 1646 significantly downregulated genes and 1165 significantly upregulated genes in *ys1 ys2-cr1* (Figure 6B). To explore which biological process was affected in *ys1 ys2-cr1*, we carried out KEGG (Kyoto Encyclopedia of Genes and Genomes) pathway enrichment analysis of the differentially expressed genes (DEGs). The results showed that the plant–pathogen interaction, phenylpropanoid biosynthesis, amino sugar and nucleotide sugar metabolism, glycerophospholipid metabolism, and porphyrin and chlorophyll metabolism were especially impacted in *ys1 ys2-cr1* (Figure 6C). Moreover, analysis of the KEGG pathway annotation classification showed that the carbohydrate metabolism changed the most in *ys1 ys2-cr1*, followed by the biosynthesis of other secondary metabolism and lipid metabolism (Figure 6D). GO (Gene Ontology) analysis consists of three categories, including the cellular component, molecular function, and the biological process. The results show that in the cellular component category, the chloroplast, mitochondrion, chloroplast stroma, chloroplast envelope, chloroplast thylakoid membrane, and thylakoid were significantly affected in *ys1 ys2-cr1*. In the molecular function category, rRNA binding, polynucleotide adenylyltransferase activity, and serine-type endopeptidase inhibitor activity were significantly affected. In the biological process category, processes such as chloroplast organization, response to wounding, response to chitin, photosynthesis, response to light stimulus, chlorophyll biosynthesis, mRNA polyadenylation, group II intron splicing, thylakoid membrane organization, and chloroplast rRNA processing were significantly affected (Figure 6E). These results indicated that YS1 and YS2 might play crucial roles in the organization of chloroplast stroma and thylakoids, as well as in chloroplast RNA processing, thus participating in photosynthesis, influencing carbohydrate metabolism, and affecting chloroplast homeostasis.

### 2.11. Carbohydrate Contents in Photosynthesis Were Decreased in ys

**Figure 7:**
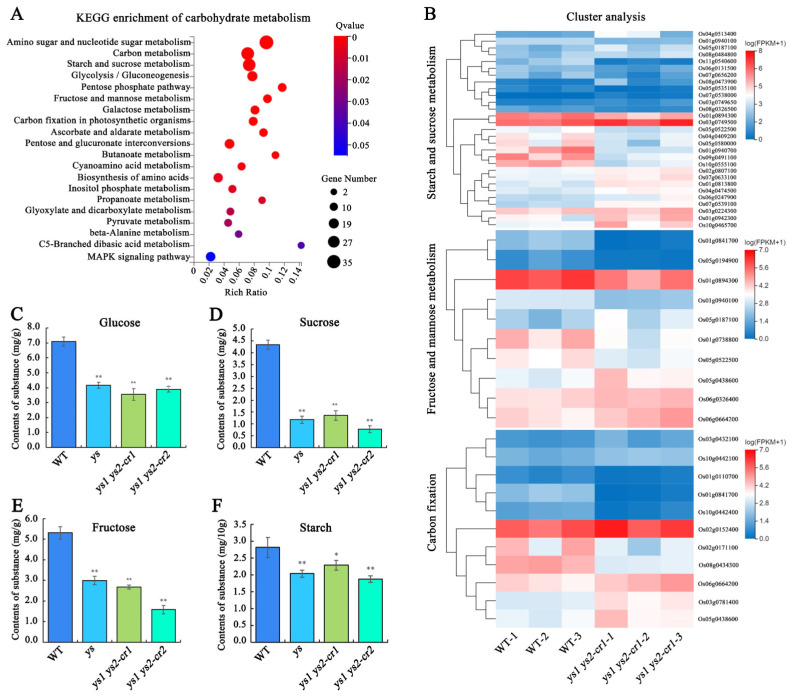
Changes of carbohydrates in 7-day seedlings of WT and *ys* mutants. (**A**) The KEGG enrichment map of carbohydrate metabolism in *ys1 ys2-cr1* compared to WT. The differential genes were analyzed based on three biological replicates with |log_2_ (fold change)| ≥ 1, Qvalue (corrected *p*-value) ≤ 0.05. (**B**) Cluster analysis of gene expression in starch and sucrose metabolism, fructose and mannose metabolism and carbon fixation in photosynthetic organisms between WT and *ys1 ys2-cr1*. (**C**) The contents of glucose in WT, *ys*, *ys1 ys2-cr1* and *ys1 ys2-cr2*. (**D**) The contents of sucrose in WT, *ys*, *ys1 ys2-cr1* and *ys1 ys2-cr2*. (**E**) The contents of fructose in WT, *ys*, *ys1 ys2-cr1* and *ys1 ys2-cr2*. (**F**) The contents of starch in WT, *ys*, *ys1 ys2-cr1* and *ys1 ys2-cr2*. The 7-day seedlings of different materials were collected and detected by the reagent kits (Solarbio). The values were calculated for three biological replicates and three technical replicates. * means *p* < 0.05; ** means *p* < 0.01.

According to the analysis presented in Figure 6D, carbohydrate metabolism in *ys1 ys2-cr1* was the most influenced in the KEGG pathway classification. To determine which type of carbohydrate metabolism was particularly affected, we carried out further analysis of carbohydrate metabolism. The results exhibited that amino sugar and nucleotide sugar metabolism, carbon metabolism, starch and sucrose metabolism, glycolysis/gluconeogenesis, pentose and glucuronate interconversions, biosynthesis of amino acids, carbon fixation in photosynthetic organisms, galactose metabolism, fructose and mannose metabolism, and the pentose phosphate pathway were significantly impacted in *ys1 ys2-cr1* (Figure 7A). This suggested that mutations in YS1 and YS2 might lead to changes in carbohydrate metabolism, especially sugar-related metabolism. Additionally, we performed cluster analysis of gene expression in starch and sugar metabolism, fructose and mannose metabolism, and carbon fixation in photosynthetic organisms. The results showed that the majority of genes in the three metabolic pathways in *ys1 ys2-cr1* were downregulated compared with those of WT (Figure 7B), indicating that the accumulation of carbohydrates in *ys1 ys2-cr1* might be inhibited.

As is well known, photosynthesis produces carbohydrates, providing energy and material for plants [33]. To investigate whether the carbohydrates produced by carbon fixation in photosynthesis in *ys* were affected, we examined the contents of glucose, sucrose, fructose, and starch in WT, *ys*, *ys1 ys2-cr1* and *ys1 ys2-cr2* seedlings at 7 DAG. Our results showed that the contents of glucose (Figure 7C), sucrose (Figure 7D), fructose (Figure 7E), and starch (Figure 7F) were significantly reduced in the *ys*, *ys1 ys2-cr1,* and *ys1 ys2-cr2* seedlings compared with the WT seedlings, indicating that the decrease in carbohydrates in *ys* mutants could be the main cause of yellowing and death of seedlings in rice. 

## 3. Discussion

### 3.1. YS1 and YS2 Participated in the Splicing of the petB Intron

PetB is a core subunit of the cytochrome b6/f complex, one of the four components of the photosynthetic system. It is also one of the electron acceptors in the chloroplast electron transport chain, which is crucial for chloroplast photosynthesis. In the *Arabidopsis prfb3* mutant, electron transport in the photosystem is hindered, and the accumulation of the cytochrome b6/f complex displays defective. Further investigation finds that PrfB3 (peptide chain release factor B3) regulates cytochrome b6 levels by stabilizing the 3′ processed *petB* transcript [34,35]. BSF (petB/petD stabilizing factor) can bind to the intergenic region of *petB-petD*, stabilizing the *petB* transcript and stimulating *petD* translation. Therefore, the mutation of *BSF* leads to reduced accumulation of the cytochrome b6/f complex protein [36]. The *petB* mutation in *Chlamydomonas* also exhibited defective assembly of the cytochrome b6f complex, an inability to conduct photosynthesis, and obstruction of photosynthetic electron transfer [37,38]. All these studies have demonstrated the significance of *petB* in chloroplasts.

Due to the degradation of intron elements, the introns lose their ability to self-splice. Therefore, the splicing of chloroplast introns is largely dependent on nuclear-coded splicing factors [8], as shown in the splicing of the *petB* intron. It was reported that OsSLA4, a PLS-type PPR protein, affects the splicing of the *petB* intron in rice, but the mechanism of its participation in the splicing is still unclear [27]. OsPPR4 is essential for seedling development, and the splicing of *petB* and *rps16* introns is partially deficient in the *osppr4* mutant [26]. In *Arabidopsis*, HCF152 is a PPR protein with 12 PPR motifs, involved in the processing of *psbB-psbT-psbH-petB-petD* transcripts. In the *hcf152* mutant, the level of spliced *petB* intron is reduced [39,40]. The CRS2 (chloroplast RNA splicing 2) and CAF2 (CRS2-associated factor 2) complexes also participate in the splicing of the *petB* transcript in *Arabidopsis* [41]. LEFKOTHEA, a nuclear-encoded PORR protein, promotes the splicing of *petB* and *rpl2* introns in *Arabidopsis*, and loss of the *LEFKOTHEA* gene exhibits etiolated seedlings and a defective chloroplast phenotype [42]. In maize, the *rnc1* (ribonuclease III domain protein 1) mutant displays deficient *petB* splicing and pale-yellow seedlings [43]. The PORR protein WTF1 (what’s this factor 1) also participates in the splicing of *petB*, and the *wtf1* mutant leads to albino seedlings in maize. Moreover, WTF1 interacts with RNC1, facilitating the splicing of the *petB* transcript [44]. In the PPR proteins OsSLA4, OsPPR4, and *Arabidopsis* HCF152, it remains unclear how OsSLA4 and OsPPR4 affect the splicing of the *petB* intron. Currently, it is known that there are severe defects in the *petB* splicing of *sla4* and partial defects in the *petB* splicing of *osppr4*, as identified using RT-PCR. HCF152 functions in the processing of *petB* by possibly stabilizing the *petB* exon–intron junctions and affects the accumulation of the spliced *petB* transcript. In our research, the *ys* mutant of rice had severe defects in the splicing of the *petB* intron and decreased levels of spliced *petB* transcript (Figure 4). Additionally, the PPR proteins YS1 and YS2 bound to the *psbH-petB* intergenic region, participating in the splicing of the *petB* intron (Figure 5E–G). Our work laid a foundation for understanding the mechanism of PPR proteins in regulating *petB* splicing in rice. However, whether YS1 and YS2 had functions similar to that of HCF152 in stabilizing the *petB* transcript remains unknown.

**Figure 8:**
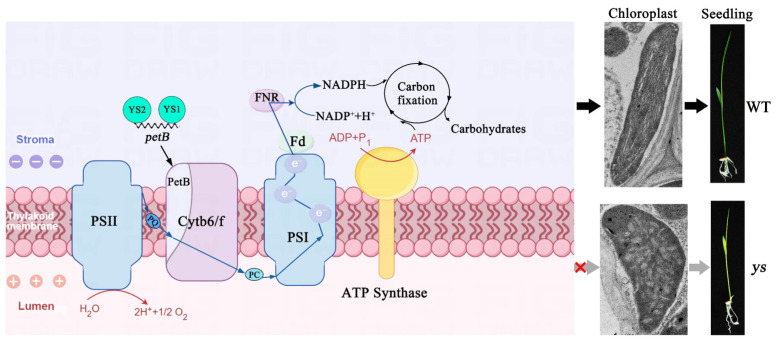
A proposed working model of YS1 and YS2 in rice. The YS1 and YS2 proteins were functionally redundant and encoded by nuclear genes, and participated in splicing of chloroplast *petB* intron, which affected the accumulation of cytochrome b6/f and PSI complexes within the photosynthetic electron transport, as well as the concentration of NADPH and the contents of carbohydrates. The mutations of YS1 and YS2 impacted on chloroplast thylakoid structure and rice seedlings development. PSII: photosystem II complex; Cyt b6/f: Cytochrome b6/f complex; PSI: photosystem I complex; Fd: ferredoxin; FNR: ferredoxin NADP^+^ oxidoreductase; PQ: plastoquinone; PC: plastocyanin.

In our study, YS1 and YS2 participated in the splicing of the *petB* intron by binding to the *psbH-petB* intergenic region (Figure 5E–G). This affected the splicing efficiency of *petD*, which is located downstream of *petB* in the five-cistronic transcription unit *psbB-psbT-psbH-petB-petD*. Therefore, the mutations in *YS1* and *YS2* resulted in decreased mature transcripts of *petB* and *petD* after splicing (Figure 4) and significantly reduced the protein level of PetB in the *ys* mutants (Figure 5H). This ultimately led to defects in the cytochrome b6/f complex and a decrease in photosynthetic activity (Figure 2D,E and Figure 3), which might be the main reasons for the reduced chlorophyll content, seedling yellowing and eventual death in *ys* (Figure 1 and Figure 8). The *ys* phenotype is similar to that of *sla4* and *ppr4* in rice, *lefkothea* in *Arabidopsis*, and *wtf1* and *rnc1* in maize. However, whether YS1 and YS2 interact with SLA4, PPR4, LEFKOTHEA, WTF1, and RNC1 in rice to regulate *petB* and whether their action sites were the same remains to be further explored.

### 3.2. YS1 and YS2 Were Essential for Functional PSI and Cytochrome b6/f Complex

Photosynthesis is one of the most significant biological processes in the biosphere, as it facilitates the assimilation of atmospheric CO_2_ using light energy and produces molecular oxygen (O_2_). The main light-driven reactions of oxygen-containing photosynthetic organisms, including cyanobacteria, algae, and plants, occur on the thylakoid membrane. These reactions are mediated by photosystem II (PSII) and photosystem I (PSI), which are coupled and work synergistically [45,46]. Electrons extracted from water via the oxygen-evolving complex (OEC) of PSII are transferred to PSI through the plastoquinone pool (PQ), the cytochrome b6/f (Cyt b6/f) complex, and plastocyanin (PC). These electrons eventually reach ferredoxin and NADP^+^, producing NADPH, which forms the electron transfer reaction chain H_2_O-PSII-PQ-Cyt b6/f-PC-PSI-NADP^+^-NADPH. The electron transfer reaction is coupled with proton pumping into the thylakoid lumen. The generated proton gradient is then used to produce ATP. Therefore, both ATP and NADPH provide fuel for carbon dioxide fixation [47,48].

The cytochrome b6/f complex coordinates electron transfer between PSI and PSII and plays a crucial role in regulating the electron transfer chain (ETC). In *Arabidopsis*, CYP37 (cyclop hilin 37) interacts with the Cyt b6/f complex subunit PetA (photosynthetic electron transfer A) to maintain the activity of the complex. The dysfunction of electron transport in *cyp37* leads to increased ROS accumulation, reduced anthocyanin biosynthesis, and enhanced chlorophyll degradation [49]. The *Arabidopsis* thylakoid proteins PGR5 (proton gradient regulation 5) and PGRL1 (pgr5-like 1) interact with each other and bind to PSI. In *pgr5* and *pgrl1* mutants, the electron transfer of photosynthesis exhibits distinct obstruction [50,51,52]. In maize, a nuclear-coded protein CRP1 (chloroplast RNA processing 1) is required for the translation of chloroplast *petA* and *petD.* The loss of *CRP1* leads to reduced accumulation of the cytochrome b6/f complex and photosystem I proteins, and the photosynthetic electron transport is also significantly affected [25,53].

In our research, the accumulation of PSII complex proteins in the *ys* mutants reached a normal level, while the Cyt b6/f and PSI complexes of the electron transfer chain were deficient (Figure 3). This might lead to dysfunction of the electron transport process and reduced production of NADPH (Figure S8), ultimately resulting in decreased fuel for carbon fixation. This is confirmed by the results showing reduced contents of glucose, sucrose, fructose, and starch, which are produced by the photosynthesis dark reaction in *ys* mutants (Figure 7C–F). The defect in the Cyt b6/f complex was undoubtedly due to the decreased levels of mature *petB* transcript and translated protein due to the impeded splicing of the *petB* intron (Figure 3, Figure 4 and Figure 5). However, the cause of the deficient PSI in *ys* remains unknown. One possibility is that the chloroplast RNA encoding the PSI protein in *ys* is abnormal. Ycf3 is essential for the PSI assembly process by interacting with the PsaA and PsaD subunits of PSI [23]. In *ys*, it seems that the amount of unspliced *ycf3-1* transcript was increased compared to that in the WT (Figure 4A), suggesting that the splicing of the *ycf3-1* intron might be partially impaired, which might be one of the reasons for damaged PSI. By comparison, the yellowing and lethal phenotype of observed in *ys* seedlings was more severe than in the *cyp37*, *pgr5*, and *pgrl1* mutants, which may be due to CYP37 acting on the Cyt b6/f complex, PGR5 and PGRL1 acting on PSI, and YS1 and YS2 acting on both the Cyt b6/f and PSI complexes. Certainly, the functional differences of these genes among different species have to be considered. It is also possible that YS1 and YS2 have other functions in different mechanisms that remain to be investigated.

### 3.3. YS1 and YS2 Were Duplicated Genes and Essential for Rice Chloroplast Development

In higher eukaryotes, gene rearrangement resulting from DNA damage repair, exchange, and transposon translocation leads to variations in chromosome structure. The main types of chromosome variations include deletion, duplication, translocation, and inversion [54,55]. Duplication, which involves adding identical fragments to chromosomes, results in the presence of duplicated genes in the relevant duplicated fragments. Segmental duplication is a major structural variation that occurs on chromosomes and is a significant source of genome evolution in eukaryotes [56]. Theoretically, segmental duplication of chromosomes leads to gene dose and compensation effects, resulting in genomic imbalances, which have been found in *budding yeast*, maize, and *Drosophila aneuploidy* [57,58,59]. Rice is a model plant, and its genome has been fully sequenced, which is propitious to the research of the global rice genome [60]. During the evolution of the rice genome, genome-wide duplication occurred about 70 million years ago. It is hypothesized that a recent fragment duplication occurred on the initial portion of chromosomes 11 and 12, approximately 5–7 million years ago [61]. However, it is also speculated that segmental duplication is actually caused by genome-wide duplication [62].

The role of duplicated genes in controlling rice development is currently unclear. A total of 491 PPR genes have been identified in the rice genome, including 246 P-type PPR and 245 PLS-type PPR genes. Homology analysis showed that 276 PPR genes are homologous to other PPR genes in rice, among which YS1 and YS2 are the most homologous [60]. Additionally, YS1 and YS2 are located within the duplicated fragments of chromosomes 11 and 12, which contain 313 pairs of duplicated genes. Among them, at least 48 pairs have similar or identical expression patterns [62]. In our study, the expression patterns of *YS1* and *YS2* were also highly similar in rice (Figure S7). However, the regulatory mechanism of duplicated genes YS1 and YS2 is unknown. Our study found that YS1 and YS2 were functionally redundant (Figure S2) and played essential roles in the structure of chloroplast thylakoids and in photosynthesis (Figure 2). Further studies discovered that YS1 and YS2 bound to the *psbH-petB* intergenic region transcript and were crucial for the splicing of chloroplast RNA, thus affecting the production of carbohydrates. Our work laid a foundation for exploring the functions of duplicated genes in higher plants.

## 4. Materials and Methods

### 4.1. Plant Materials and Growth Conditions

The rice (*Oryza sativa* L.) *ys* (*yellow-green seedling*) mutant without the insertion of T-DNA was obtained from the RISE DB (Rice T-DNA Insertion Sequence Database). The *ys* mutant was cloned using a sequencing-based mapping method [28]. The single gene knockdown mutants *ys1-cr* and *ys2-cr* as well as the double gene knockdown mutants *ys1 ys2-cr* were obtained using CRISPR/Cas9 technique [63]. The target sites were designed on the CRISPR direct (https://crispr.dbcls.jp/) and cloned into CRISPR/Cas9 vector. The plasmids were transformed into EHA105 (*Agrobacterium tumefaciens*), and then were infected into rice (*Oryza Sativa* L. spp. *japonica*) by the callus infection method [64]. Seeds of wild type (WT), *ys*, *ys1-cr*, *ys2-cr* and *ys1 ys2-cr* were germinated on 1/2 MS medium containing 3% (*w*/*v*) sucrose, and cultivated in the greenhouse of Wuhan University under 30 ± 2 °C and in the natural environment of outdoor square (March to October).

### 4.2. Measurement of Chlorophyll and Carotenoid Contents

We measured the contents of chlorophyll a, chlorophyll b and carotenoid in WT, *ys* and *ys1 ys2-cr1* according to the method as the reported [65]. The 0.2 g fresh leave samples were harvested from seedlings at 5, 7 and 10 days after germination (DAG), respectively, and frozen in the liquid nitrogen. Total pigments were extracted with 1 mL 96% ethanol per 0.02 g sample and placed in the dark for 10 h. The mixtures then were centrifuged at 14,000× *g* and 4 °C for 10 min and supernatants were used to detect the contents of pigments. The absorbance values were detected at 470 nm, 649 nm and 665 nm wavelengths by spectrophotometer, with the extraction solution of pigment as control. The values were calculated based on three biological replicates and three technical replicates.

### 4.3. Transmission Electron Microscopy (TEM)

We observed the subcellular organelle structures by transmission electron microscopy (TEM) as the previously described [66]. The 7 DAG leaves in WT, *ys* and *ys1 ys2-cr1* were fixed in the pre-fixative solution with 2% glutaraldehyde and in the post-fixative solution with 1% osmic acid, dehydrated in gradually increasing alcohol gradients, embedded in Spurr resin, sections and stained with lead citrate, and then observed under TEM.

### 4.4. Detection of Photosynthetic Efficiency

The Chlorophyll fluorescence parameters (Fv/Fm) were measured as reported [67]. The 7 DAG leaves of WT, *ys* and *ys1 ys2-cr1* seedlings were cut and placed successively in square petri dishes containing 1/2 MS medium after 30 min dark adaption. The Chlorophyll fluorescence imaging system Fluor Cam800MF (Photon Systems Instruments Ltd., Brno, Czech Republic) was debugged by adjusting the activity button to 25%, super button to 50%, and sensitivity button to 17–21% in the Global light setting. The measurement was conducted with three biological replicates.

### 4.5. Protein Isolation and Immunoblot Analysis

Total proteins were extracted referring to the reported method [63]. The 7 DAG leaves in WT, *ys*, *ys1 ys2-cr1* and *ys1 ys2-cr2* were respectively collected, and their total proteins were extracted with the lysis buffer (0.5% NP40, 1 mM PMSF, 1×protease inhibitor cocktail-CWBIO CW2200S, 150 mM NaCl and 100 mM Tris-HCl PH7.5). The protein concentration was determined by the BCA protein quantitative reagent kit (Dingguo Changsheng Biotechnology Co., BCA02, Beijing, China), and the sample loading amount was 10 μg. The total proteins were separated by SDS-PAGE gel, transferred on activated cellulose acetate membrane, and incubated with primary antibodies (anti-photosystem proteins antibodies were from Hou Lab as described in previous report, and anti-mitochondrial protein antibodies were from Proteintech) [68] and secondary antibodies (ABclonal Biotechnology Co., Wuhan, China), respectively. The protein bands were detected using HRP chemiluminescence kit (Yeasen Biotechnology Co., S8315210, Shanghai, China) by the protein scanning instrument Amersham Imager 680 (GE Healthcare Bio-Sciences AB, Uppsala, Sweden).

### 4.6. Phylogenetic Analysis

The amino acid sequences of rice YS1 (LOC_Os11g01210) and YS2 (LOC_Os12g01210) were downloaded from RGAP (Rice Genome Annotation Project) website (http://rice.uga.edu), and input into the NCBI BLAST website (Available online: https://blast.ncbi.nlm.nih.gov/Blast.cgi (accessed on 20 September 2024)) to search for homologous proteins in different species. We aligned rice YS1 and YS2 with homologous sequences in other species with ClustalX (http://www.clustal.org/), and then constructed the phylogenetic tree with MEGA7.0 by neighbor-joining method.

### 4.7. PPR Motif Alignment Among Homologous Proteins

The homologous protein sequences of YS1 and YS2 in monocotyledons downloaded from NCBI were aligned by multiple sequences using DNAMAN software (https://www.lynnon.com/), and the more similar sequences were marked with darker colors. We uploaded these sequences to the PPR website (https://ppr.plantenergy.uwa.edu.au), selected plant-specific comparison, and obtained the position and number of PPR motif on these sequences. We plotted the PPR motif characteristics on these sequences in Photoshop 2022 software.

### 4.8. Total RNA Extraction and RT-PCR

Total RNAs from 7 DAG rice leaves of WT and mutants were extracted with *TransZol* plant kit (TransGen Biotech., ET121, Beijing, China), and reverse-transcribed into cDNA by the kit of TransGen reverse transcription (TransGen Biotech., AE311, Beijing, China). The Random primers were used for reverse transcriptions to detect the intron splicing by semi-quantitative RT-PCR, and the Oligo dTs were used for reverse transcription to detect splicing efficiencies of transcripts by RT-qPCR. We performed the RT-qPCR by *TransStart* Top Green qPCR SuperMix kit (TransGen Biotech., AQ131, Beijing, China) using Bio-Rad CFX Manager 3.1 instrument (Bio-Rad Laboratories Inc., Singapore), and selected *Actin* and *Ubiquitin* as two reference genes.

### 4.9. Prokaryotic Protein Expression, Purification, and RNA Electrophoretic Mobility Shift Assays (REMSA)

We cloned the encoding sequences of YS1 and YS2 onto PET28b vector that carried the MBP tag. The YS1-MBP and YS2-MBP plasmids were transformed into Transetta (DE3) Chemically Competent Cell (TransGen Biotech., CD601, Beijing, China) and induced at 28 °C with 0.5 mM IPTG to express the prokaryotic proteins. The two bacterial solutions were broken in high-pressure cell disrupter, the supernatants were collected and combined with MBP agarose beads, and then eluted to obtain the purified proteins.

The FAM-labeled RNA probe was co-incubated with different concentrations of YS1-MBP and YS2-MBP proteins in the binding buffer (1 U/μL RNAase inhibitor, 5 mM MgCl_2_, 25 mM Tris-HCl pH 8.0, 40 mM NaCl, 2% glycerol) at 25 °C for 30 min, with blank protein as the negative control and 6 times unlabeled competitive probe and non-competitive probe as the experimental controls. The RNA-protein complex was isolated in 6% undenatured polyacrylamide gel and scanned at an excitation wavelength of 492 nm under the protein scanning instrument Amersham Imager 680 (GE Healthcare Bio-Sciences AB, Uppsala, Sweden).

### 4.10. Detection of NADPH Concentration

The 7 DAG seedlings of WT, *ys*, *ys1 ys2-cr1* and *ys1 ys2-cr2* were used for detecting NADPH concentration by the amplite™ fluorimetric NADPH assay kit (AAT Bioquest Inc., Cat No. 15262, Pleasanton, CA, USA). We prepared the NADPH standard solution with different concentrations and NADPH working solution, and then generated the standard curve. The process was operated in the dark on ice. The rice samples were pre-treated according to the manual of kit and incubated with NADPH working solution at room temperature for 30 min. Afterwards, the fluorescence values were measured under 540 nm excitation light and 590 nm emission light.

### 4.11. Transcriptome Sequencing and Analysis

The 7 DAG WT and *ys1 ys2-cr1* seedlings were collected, and cDNA libraries were constructed and sequenced on DNBSEQ at BGI Genomics. The sequencing was conducted with three biological replicates. The average mapping ratio between the sequencing data and rice reference genome from RGAP was 95.23%. Sample correlation was calculated by the Pearson correlation coefficient of all gene expression levels between two samples. The correlation coefficient could reflect the similarity of overall gene expression among different samples. Differentially expressed genes (DEGs) between WT and *ys1 ys2-cr1* mutants were analyzed by DEseq2 (Available online: http://bioconductor.org/packages/stats/bioc/DESeq2 (accessed on 5 September 2023)) based on the FPKM with the adjusted *p*-value ≤ 0.05, and the |log_2_ (fold change)| ≥ 1. The KEGG and GO analysis of DEGs were performed with BGI technology platform.

### 4.12. Determination of Sugars and Starch Contents

The 7-day seedlings of WT, *ys*, *ys1 ys2-cr1* and *ys1 ys2-cr2* were collected for detecting contents of glucose, sucrose, fructose, and starch. The glucose contents were determined using glucose test kit (Solarbio Science & Technology Co., BC2505, Beijing, China). Glucose was oxidized to gluconic acid under the catalysis of glucose oxidase (GOD), producing hydrogen peroxide. The hydrogen peroxide was catalyzed by peroxidase (POD) to oxidize 4-amino-antipyrine conjugated phenol and produced colored compounds with maximum absorption peaks at 505 nm. The sucrose contents were determined using sucrose test kit (Solarbio Science & Technology Co., BC2465, Beijing, China). Sucrose was hydrolyzed to produce glucose and fructose, and the fructose further reacted with resorcin to produce colored substance with a maximum absorption peak at 480 nm. The fructose contents were determined using fructose test kit (Solarbio Science & Technology Co., BC2455, Beijing, China). The measurement of fructose was similar to that of sucrose, except that the standard substance was different. The starch contents were determined using starch test kit (Solarbio Science & Technology Co., BC0705, Beijing, China). The starch in samples could be separated with soluble sugars by 80% ethanol, further decomposed into glucose by acid hydrolysis, and detected by anthrone colorimetric method. The values were calculated based on three biological replicates and three technical replicates. The differences in contents between WT and *ys* mutants were calculated with DPS7.0 software.

## Data Availability

The relevant data that support the findings are available in the supplemental information of this article.

## Supplementary Materials

The following supporting information can be downloaded at: https://www.mdpi.com/article/10.3390/ijms26094459/s1.
