# Supplementary material for: P-Type Pentatricopeptide Repeat Proteins YS1 and YS2 Function in Splicing of petB Intron to Maintain Chloroplast Homeostasis During Rice Seedling Development
Source: Int J Mol Sci. 2025 May 7;26(9):4459. doi: 10.3390/ijms26094459 (PMC12072890; doi:10.3390/ijms26094459)
Supplement: Supplementary file 1 [file ijms-26-04459-s001.zip › ijms-3569700-supplementary.pdf]

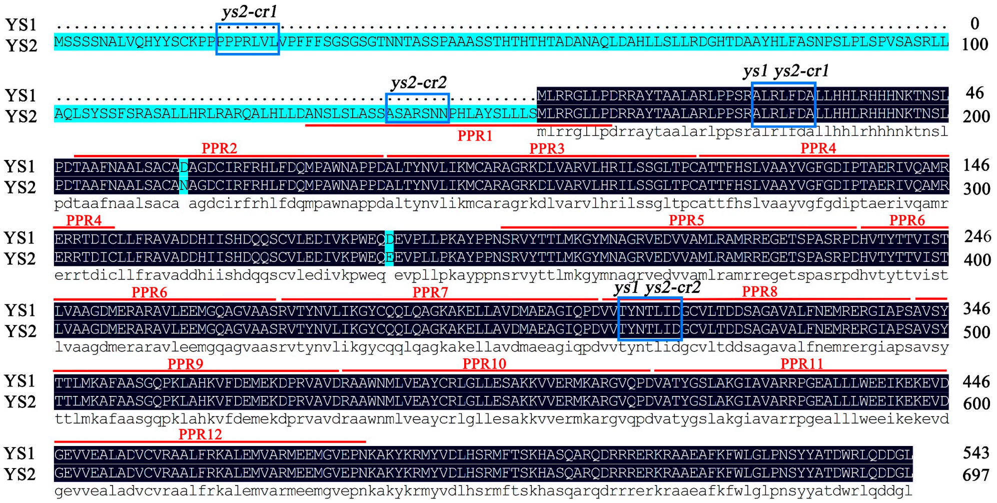

Supplemental Figure S1. Amino acid sequence alignment and structural domain characteristics of YS1 and YS2 in rice. The amino acid sequences of YS1 and YS2 were downloaded from RGAP (Rice Genome Annotation Project). The two sequences were aligned on DNAMAN software. The blue boxes marked the target sites of CRIPSR/Cas9 vector, and the red lines marked the position of the PPR motifs on YS1 and YS2 sequences.

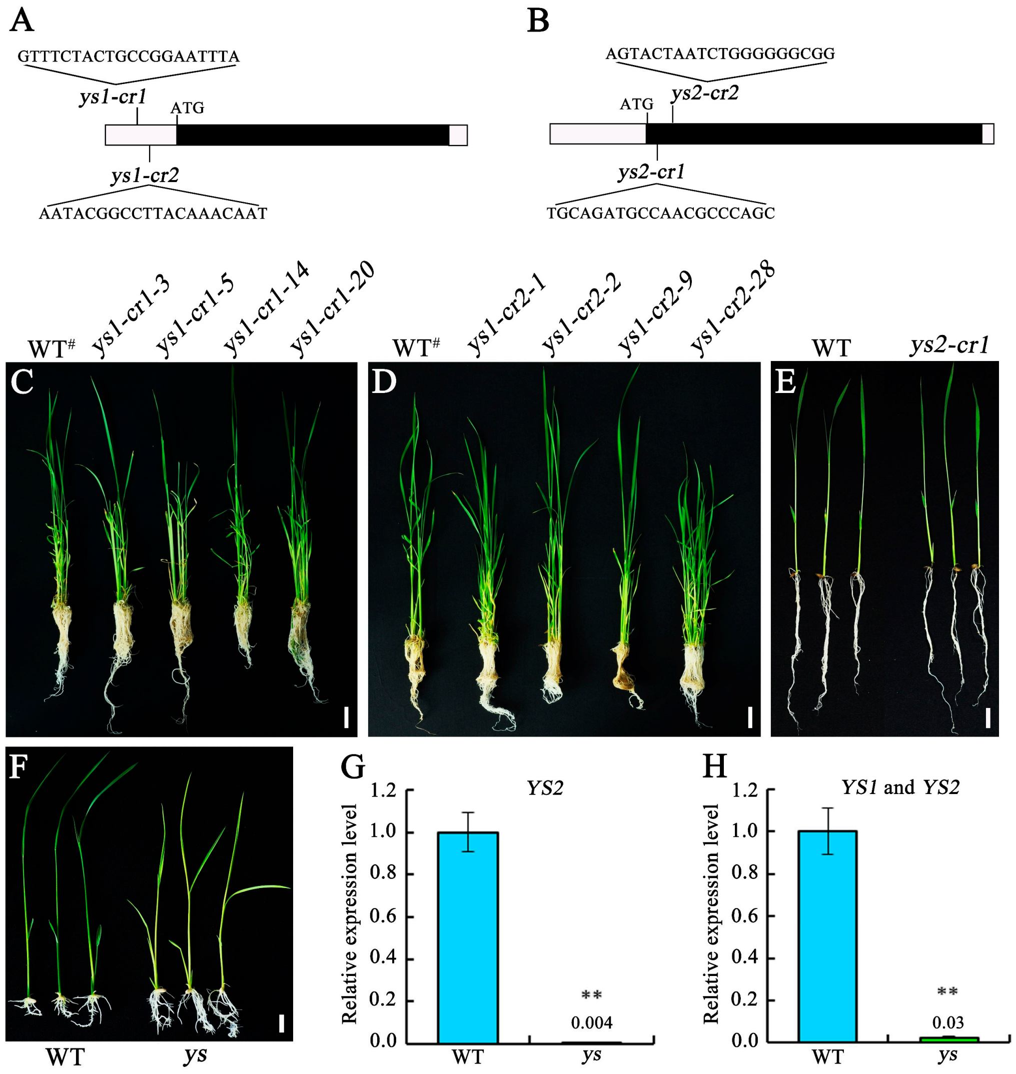

Supplemental Figure S2. Phenotypic characteristics of *ys1-cr* and *ys2-cr* mutants and the expression levels of rice *YS1* and *YS2* in *ys*. **(A)** Schematic diagram of *ys1-cr* target sites. **(B)** Schematic diagram of *ys2-cr* target sites. The black and white boxes represent exon and UTRs, respectively. **(C, D)** Phenotype of *ys1-cr1* and *ys1-cr2* seedlings at T_0 generation. WT# represents wild type rice that had undergone the transgenic process but its genome had not been edited. **(E)** Phenotype of *ys2-cr1* seedlings at T_2 generation. **(F-H)** The 7 DAG seedlings of *ys*, and the RT-qPCR of *YS2* and *YS1 YS2* expression levels in *ys* seedlings at 7 DAG. Scale bars in pictures C and D = 2 cm, and scale bars in pictures E and F = 1 cm. The values were calculated for three biological replicates and three technical replicates. ** means P<0.01.

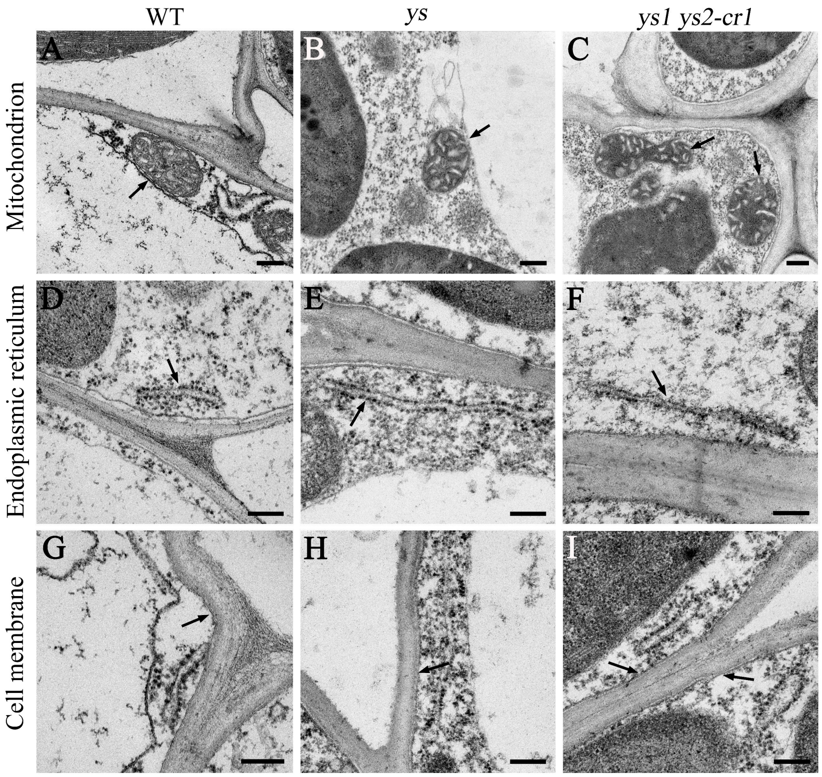

Supplemental Figure S3. Ultrastructure of subcellular organelles in 7 DAG leaves in WT, *ys* and *ys1 ys2-cr1*. **(A-C)** Mitochondria. **(D-F)** Endoplasmic reticulum. **(G-I)** Cell membrane. The ultrastructure of mitochondria, endoplasmic reticulum and cell membrane in *ys* and *ys1 ys2-cr1* were regular and ordered, and no difference with WT. The black arrows showed the position of organelles. Scale bars = 0.5 mm.

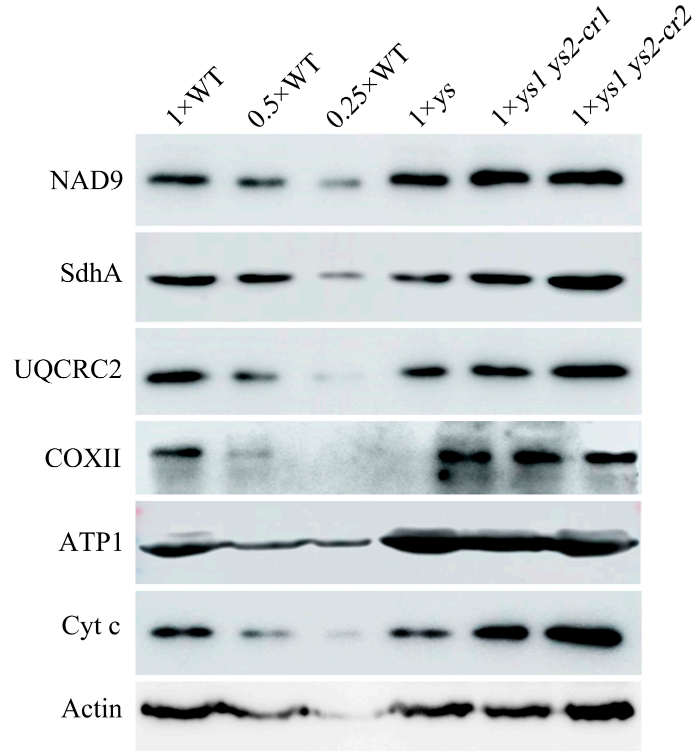

Supplemental Figure S4. Immunoblot analysis of mitochondrial complex proteins in WT, *ys*, *ys1 ys2-cr1* and *ys1 ys2-cr2* of rice. The NAD9 is respiratory chain complex I subunit; SdhA is complex II subunit; UQCRC2 is complex III subunit; COXII is complex IV subunit; ATP1 is complex V subunit; Cyt c is the electron acceptor. Actin was used as an internal reference. Primary antibodies against respiratory chain complex proteins were the Goat anti-Rabbit antiserums, and primary antibodies against Cyt c and actin were the Goat anti-Mouse antiserums.

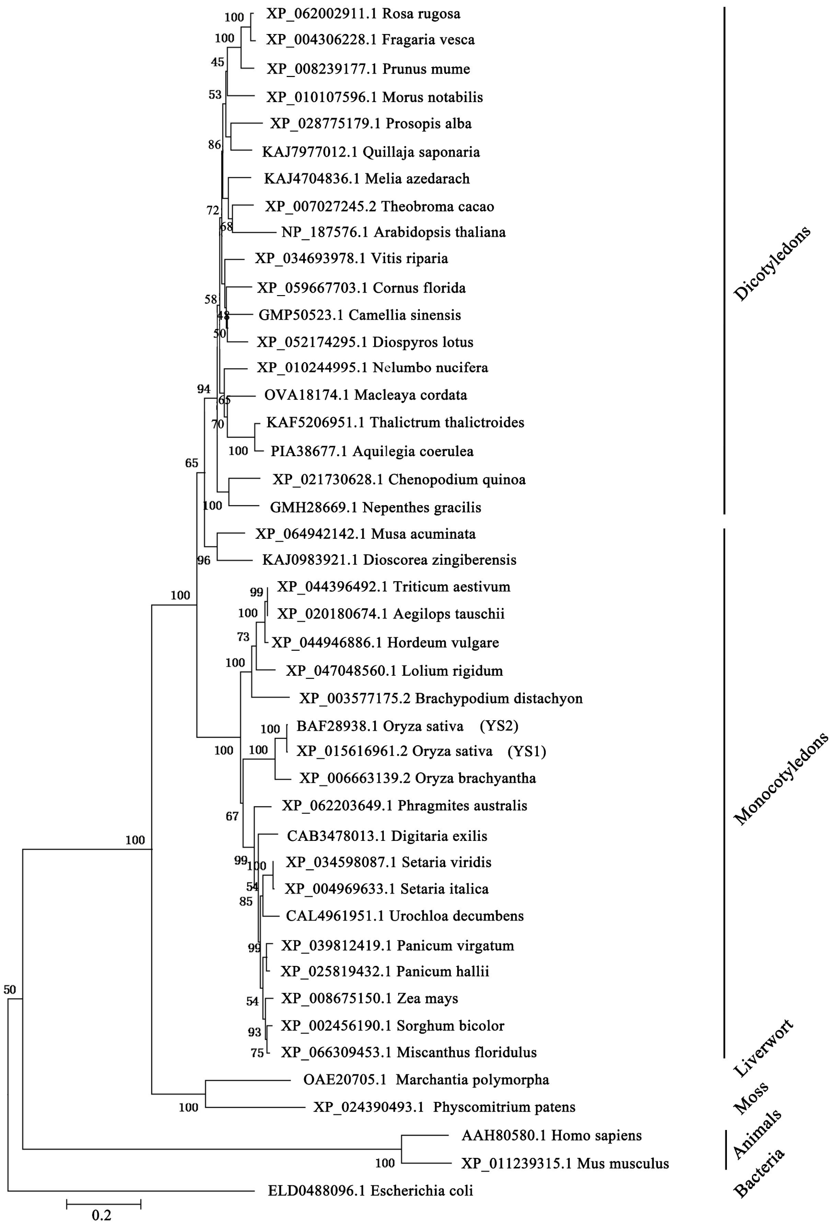

Supplemental Figure S5. Phylogenetic tree analysis of YS1 and YS2 in different species. The protein sequences of rice YS1 and YS2 were downloaded from RGAP (Rice Genome Annotation Project), and homologous sequences of rice YS1 and YS2 in other 42 species were downloaded from NCBI. The 44 sequences were aligned on ClustalX software. MEGA7.0 software was used for constructing phylogenetic tree by neighbor-joining method.

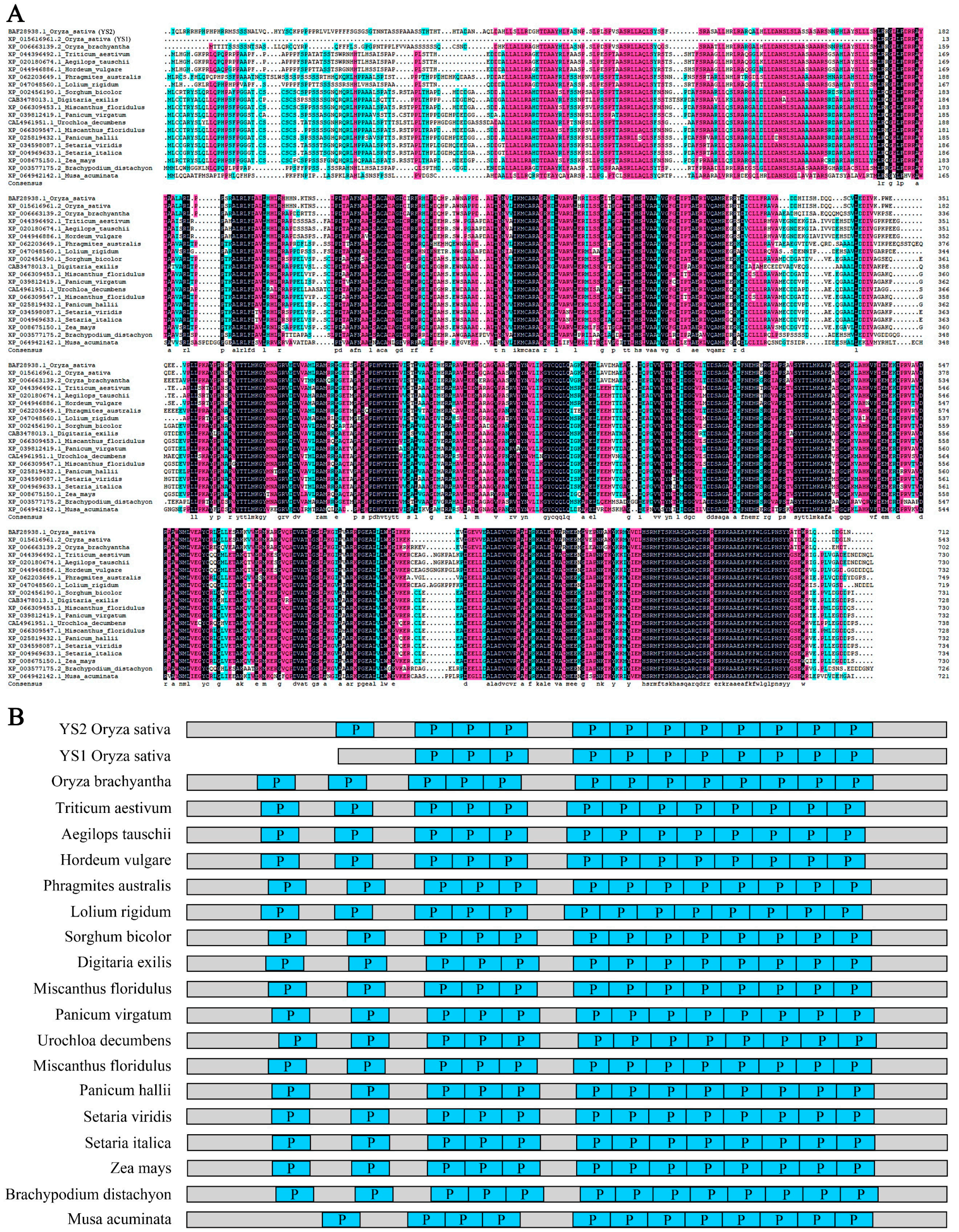

Supplemental Figure S6. Homologous alignment and structure diagrams of YS1 and YS2 in monocotyledons. **(A)** Homologous alignment of YS1 and YS2 in monocotyledons. Homologous protein sequences of YS1 and YS2 in 19 species from monocotyledons were downloaded from NCBI and aligned on DNAMAN software. The darker color on sequences, the higher similarity in the sequences. **(B)** Structure diagrams of YS1 and YS2 in monocotyledons. Domains on the sequences of YS1 and YS2 in 19 species from monocotyledons were predicted on the PPR website. The light blue boxes represent PPR motifs and the "P" represents P-type PPR motif.

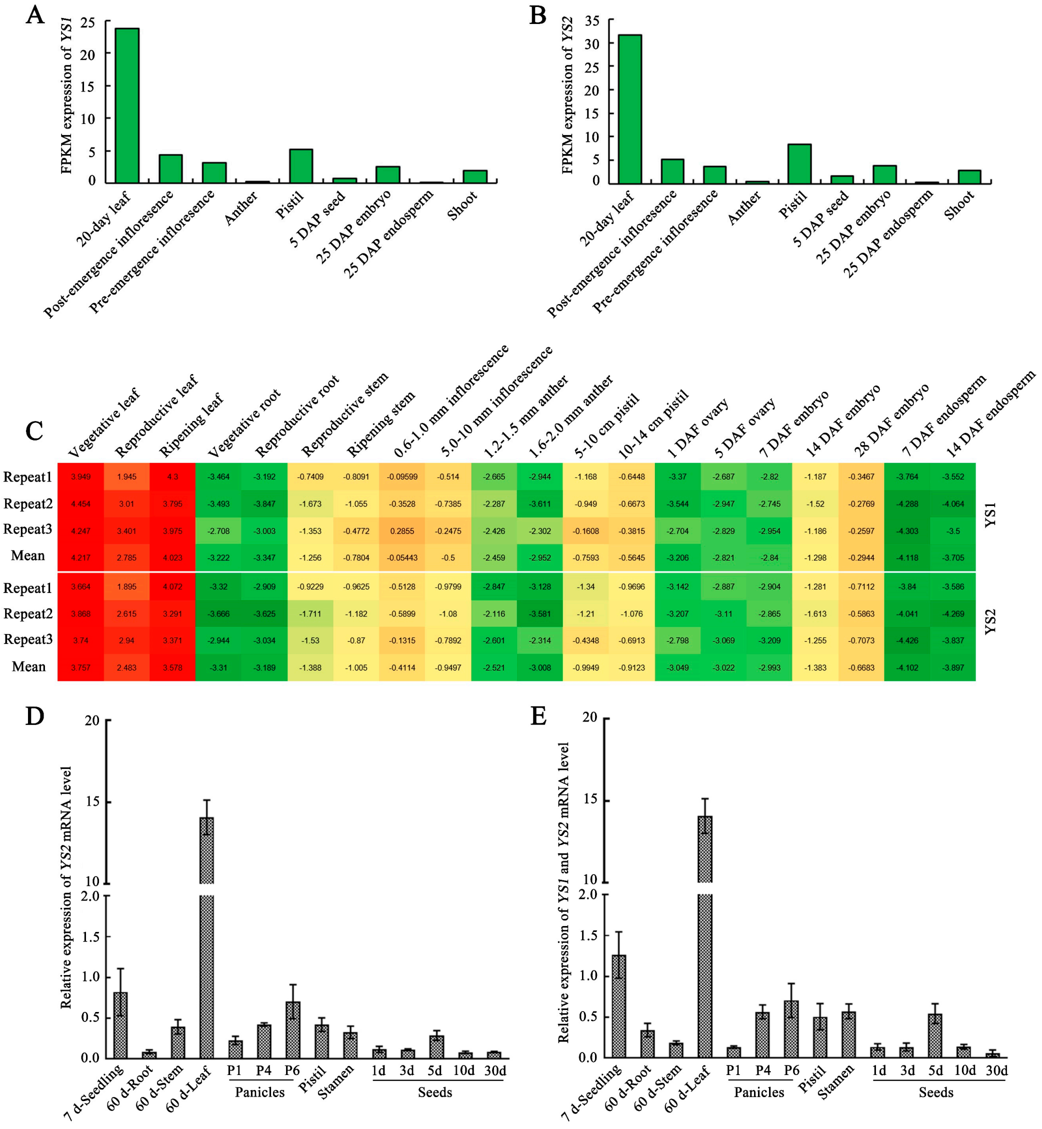

Supplemental Figure S7. Expression levels of *YS1* and *YS2* in different tissues of WT rice. **(A)** RNA-seq FPKM expression of YS1. **(B)** RNA-seq FPKM expression of YS2. **(C)** Microarray heat maps of *YS1* and *YS2* in specific tissues and organs at different developmental stages. The expression profiles were shown as normalized data (log2) of Cy3 signal strength. **(D)** The mRNA level of *YS2* in different tissues. **(E)** The mRNA levels of *YS1* and *YS2* in different tissues. 7 d-Seedling: seedling at 7 days after germination (DAG); 60 d-Root: root of 60 DAG rice plant; 60 d-Stem: stem of 60 DAG rice plant; 60 d-Leaf: leaf of 60 DAG rice plant; P1: panicle at 0-3 cm; P4: panicle at 10-15 cm; P6: panicle at 22-30 cm. *Ubiquitin* and *Actin* were used as two internal reference genes. The values were calculated for three biological replicates and three technical replicates.

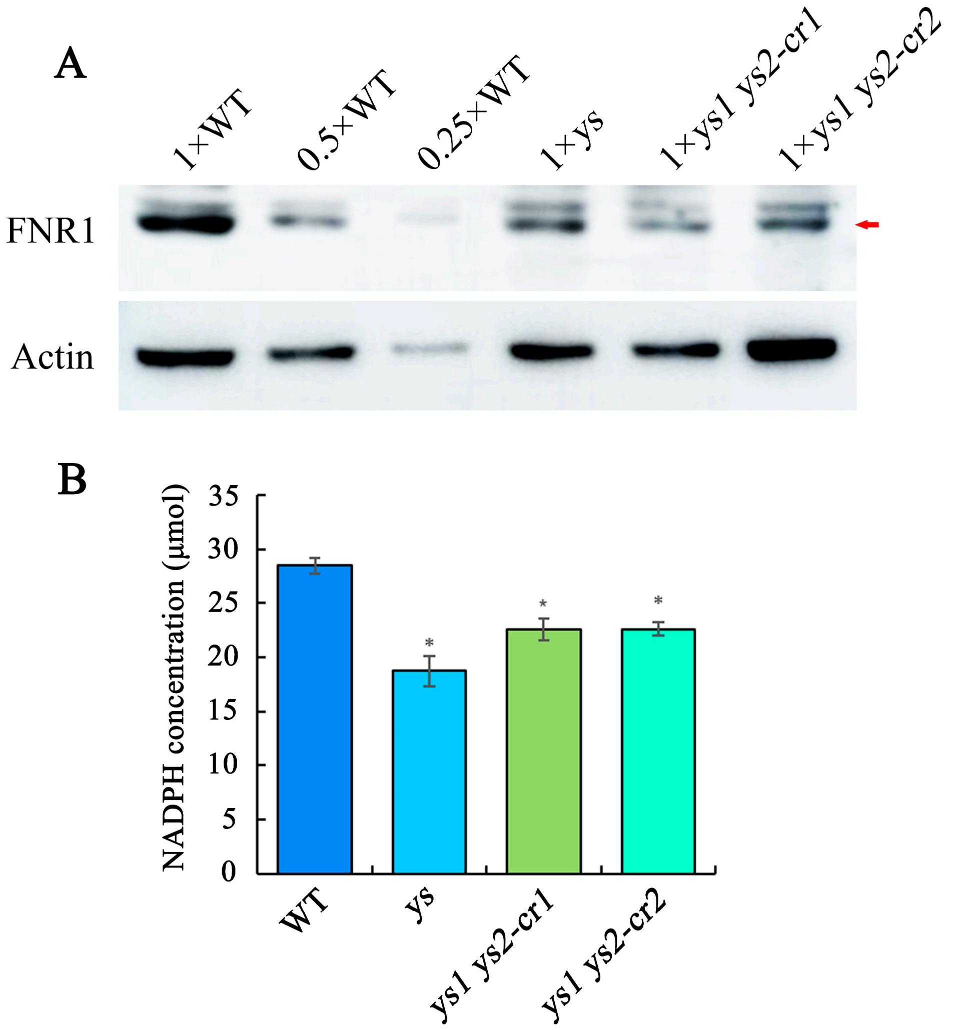

Supplemental Figure S8. FNR1 protein levels and NADPH concentrations in WT and *ys* mutants. **(A)** The expression levels of FNR1 protein in WT, *ys*, *ys1 ys2-cr1* and *ys1 ys2-cr2* leaves at 7 DAG. Primary antibody against FNR1 used in the immunoblot was the Goat anti-Rabbit antiserum. Actin as an internal reference protein. **(B)** NADPH concentrations in WT, *ys*, *ys1 ys2-cr1* and *ys1 ys2-cr2* seedlings at 7 DAG. The values were calculated for three biological replicates and three technical replicates. * means P<0.05.
